# Bee Venom Phospholipase A2 Protects against Acetaminophen-Induced Acute Liver Injury by Modulating Regulatory T Cells and IL-10 in Mice

**DOI:** 10.1371/journal.pone.0114726

**Published:** 2014-12-05

**Authors:** Hyunseong Kim, Dong June Keum, Jung won Kwak, Hwan-Suck Chung, Hyunsu Bae

**Affiliations:** 1 Department of Physiology, College of Korean Medicine, Kyung Hee University, 1 Hoeki-Dong, Dongdaemoon-gu, Seoul 130-701, Republic of Korea; 2 Institute of Korean Medicine, Kyung Hee University, 1 Hoeki-Dong, Dongdaemoon-gu, Seoul 130-701, Republic of Korea; Universidad de Costa Rica, Costa Rica

## Abstract

The aim of this study was to investigate the protective effects of phospholipase A2 (PLA2) from bee venom against acetaminophen-induced hepatotoxicity through CD4^+^CD25^+^Foxp3^+^ T cells (Treg) in mice. Acetaminophen (APAP) is a widely used antipyretic and analgesic, but an acute or cumulative overdose of acetaminophen can cause severe hepatic failure. Tregs have been reported to possess protective effects in various liver diseases and kidney toxicity. We previously found that bee venom strongly increased the Treg population in splenocytes and subsequently suppressed immune disorders. More recently, we found that the effective component of bee venom is PLA2. Thus, we hypothesized that PLA2 could protect against liver injury induced by acetaminophen. To evaluate the hepatoprotective effects of PLA2, C57BL/6 mice or interleukin-10-deficient (IL-10^−/−^) mice were injected with PLA2 once a day for five days and sacrificed 24 h (h) after acetaminophen injection. The blood sera were collected 0, 6, and 24 h after acetaminophen injection for the analysis of aspartate aminotransferase (AST) and alanine aminotransferase (ALT). PLA2-injected mice showed reduced levels of serum AST, ALT, proinflammatory cytokines, and nitric oxide (NO) compared with the PBS-injected control mice. However, IL-10 was significantly increased in the PLA2-injected mice. These hepatic protective effects were abolished in Treg-depleted mice by antibody treatment and in IL-10^−/−^ mice. Based on these findings, it can be concluded that the protective effects of PLA2 against acetaminophen-induced hepatotoxicity can be mediated by modulating the Treg and IL-10 production.

## Introduction

Acetaminophen is an effective antipyretic and analgesic drug that is commonly used. It is considered safe at its therapeutic dose, but it can cause severe hepatic necrosis, nephrotoxicity, additional hepatic lesions, and even death in experimental mice and humans when taken in high doses [1], [2]. Many researchers have attempted to demonstrate the mechanism underlying acetaminophen-induced acute injury, particularly the signaling pathways leading to tissue damage and toxicity in the liver [3], [4], [5], [6].

Tregs have been known to play a pivotal role in the maintenance of tolerance in the immune system, and Treg deficiency can be a cause of autoimmune disease [7]. Tregs also have various functions in the control of transplantation tolerance, tumor immunity, allergy, and infection [8], [9], [10].

Previous studies demonstrated that Tregs mediate therapeutic potential against immune-mediated hepatic injury [11], [12], [13]. The expression of anti-inflammatory factors, such as IL-10, has been found to be increased in the normal response to drug-induced liver injury [14]. The increased susceptibility to acetaminophen-induced hepatic injury appeared to be correlated with an elevated expression of proinflammatory cytokines, such as TNF and IL-6 [15].

PLA2 is known to be a major component of snake venoms and hydrolyzes the fatty acids in membrane phospholipids [16]. PLA2 from bee venom is a prototypic group III enzyme that hydrolyzes fatty acids, and it has been reported that melittin in bee venom enhances the activity of PLA2 [17], [18]. In addition, it has been demonstrated that this bee PLA2 prevents neuronal cell death and spinal cord injury [19], [20]. In this study, we demonstrate that PLA2 protects against hepatic dysfunction and induces antiinflammatory cytokine production in acetaminophen-injected mice by upregulation of the Treg population. Therefore, PLA2 may have therapeutic potential in preventing acetaminophen-induced hepatotoxicity.

## Materials and Methods

### Mouse

Male C57BL/6 mice (seven to eight weeks old, Charles River Korea, Seungnam, Korea), weighing 20–21 g each, were used in most of the experiments. Male Foxp3^EGFP^C57BL/6 mice (C. Cg-*Foxp3^tm2Tch^*/J, six weeks old) and IL-10^−/−^ mice (B10.129P2(B6)-*Il10^tm1Cgn^*/J, 7–8 weeks old) were purchased from The Jackson Laboratory (Bar Harbor, ME, USA). The mice were maintained under specific pathogen-free conditions with an air conditioning system and a 12-h light/12-h dark cycle. The mice had free access to food and water during the experiments. This research was approved by the Animal Care and Use Committee of Kyung Hee University (KHUASP (SE)-11-041). Mice were sacrificed by CO2 asphyxiation.

### Chemicals and treatment

PLA2 from honey bee venom and acetaminophen were purchased from Sigma-Aldrich (St. Louis, MO, USA). Before the acetaminophen injection, the mice were intraperitoneally injected with PLA2 at a concentration of 0.2 mg/kg body weight once a day for five days. The control group received an equal volume of PBS. Acetaminophen was dissolved in PBS at a concentration of 20 mg/ml. Two days after the last administration of PLA2 or PBS, all of the mice received a single intraperitoneal (i.p.) injection of acetaminophen (500 mg/kg). The mice were sacrificed by CO2 asphyxiation 24 h after the acetaminophen injection. Blood, spleen, and liver samples were obtained for further analysis.

### Flow cytometry analysis

Splenocytes were isolated from Foxp3^EGFP^ mice for analysis of the Treg population change by PLA2 treatment. The cells were treated with PBS or PLA2 (0.01, 0.1, 1 and 10 µg/ml) and cultured in complete RPMI 1640 media containing 2.5 µg/ml anti-mouse CD3 antibody and 2 µg/ml anti-mouse CD28 antibody for 72 h. The cells were incubated with fluorescently tagged Abs for CD4 and CD25 staining (eBioscience, San Diego, CA, USA). The FACS data were acquired with a FACS Calibur flow cytometer (BD Biosciences, San Jose, CA, USA), and the data were analyzed by Cell Quest Pro (BD Biosciences, San Jose, CA, USA).

### Assessment of serum AST, ALT and IL-10

Blood samples were collected 0, 6, and 24 h after the acetaminophen injection for the measurement of hepatic dysfunction by quantification of AST and ALT. The blood samples were maintained at room temperature for 1 h and then centrifuged for 10 min at 1,000 *g* to separate the serum. The AST and ALT levels were measured using a Fuji Dri-Chem 3500i instrument (Fuji Photo Film Ltd., Tokyo, Japan). The serum IL-10 level was measured by ELISA (BD Biosciences, San Jose, CA, USA).

### H&E staining

The separated livers were fixed in 4% paraformaldehyde (PFA) for 1 day and then embedded in paraffin. The paraffin samples were sliced into 5-µm-thick slices and then deparaffinized. To observe the tissues, we stained the samples in hematoxylin for 90 s and dipped then slowly three times in eosin. After washing for 10 min in running water, the samples were covered with a cover glass. The portal and periportal areas in the liver were captured by microscopy.

### Injection of anti-CD25 antibody for Treg depletion

Anti-mouse CD25 rat IgG1 (anti-CD25; clone PC61) antibody was generated from hybridomas collected from ATCC (Manassas, VA, USA). To deplete Tregs, an anti-CD25 antibody (0.1 mg/mouse) was injected i.p. each day before the PLA2 and acetaminophen injections. The depletion of Tregs was confirmed by flow cytometry analysis using PE-anti-mouse CD25 and FITC-anti-mouse CD4 antibodies.

### Assessment of proinflammatory cytokines and nitrite in the liver

Separated livers were maintained in a deep freezer (−70°C) to measure liver tissue inflammation after acetaminophen injection. Frozen liver tissues were homogenized in a protein extraction solution (PRO-PREP; Intron biotechnology, Sungnam, Korea), incubated for 30 min on ice and then centrifuged at 13,000 rpm (4°C) for 10 min. The TNF and IL-6 protein levels in the liver were measured by an enzyme linked immunosorbent assay (ELISA; BD Biosciences, San Jose, CA, USA). To measure the nitrite levels, the samples were incubated with an equal volume of Griess reagent (1% sulfanilamide/0.1% N-(1-naphthyl)-ethylenediamine dihydrochloride/2.5% H_3_PO_4_) at room temperature for 10 min. The protein concentrations of the samples were measured by a BCA^TH^ Protein Assay Kit (Thermo Scientific, Rockford, IL, USA). The final concentrations were calculated with the total amount of protein, and the results are expressed as pg/mg or pmol/mg.

### Statistical analysis

All of the results are expressed as the means ± S.E.M. The data were analyzed using two-tailed *t* test or one-way ANOVA with Tukey's test. Differences were considered to be significant at *p*<0.05.

## Results

### Upregulation of the Treg population in splenocytes by PLA2

**Figure 1:**
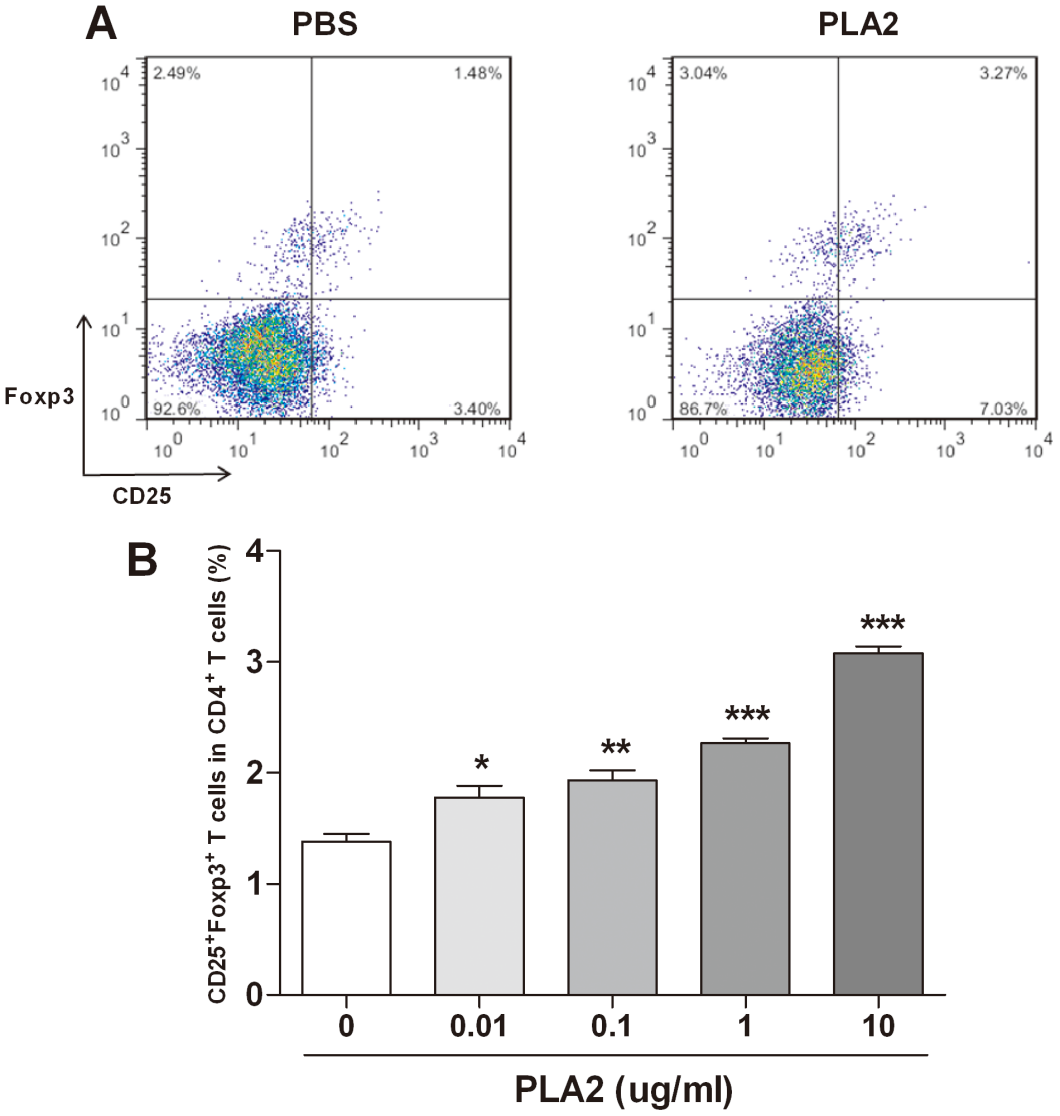
Increase of the Treg population in splenocytes by PLA2. Splenocytes from Foxp3^EGFP^ mice were treated with various concentrations of PLA2 and PBS for three days. The flow cytometry data showed a population of Tregs in the groups of PBS-treated and PLA2 (10 µg/ml)-treated CD4^+^ T cells (A). The populations of CD25^+^Foxp3^+^ T cells treated with various concentrations of PLA2 are depicted as percentages of the total CD4^+^ T cells (B). The values shown indicate the means ± S.E.M. *P<0.05 vs. PBS, **P<0.01 vs. PBS, ***P<0.01 vs. PBS.

To evaluate the immune-modulating effect of PLA2 in splenocytes, the splenocytes from Foxp3^EGFP^ mice were treated with PLA2 or PBS for three days. The Treg population was dose-dependently increased in the PLA2-treated group compared with the PBS-treated group (Fig. 1).

### Protective effects of PLA2 on acetaminophen-induced hepatotoxicity

**Figure 2:**
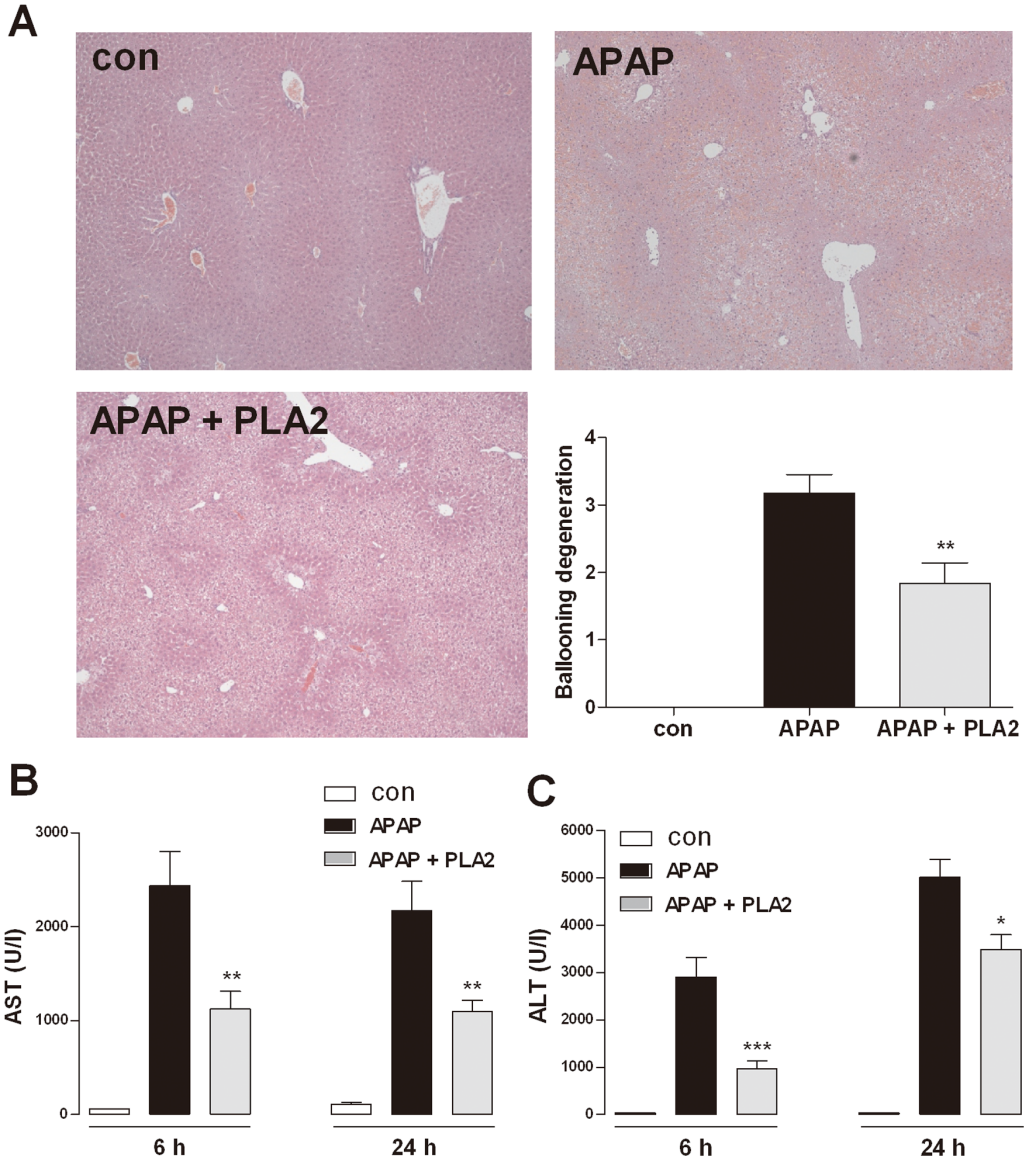
Protective effects of PLA2 on acetaminophen-induced hepatotoxicity. Mice were administered PLA2 (0.2 mg/kg) once a day for five days. The control group received the same volume of PBS. After the fifth administration of PLA2 or PBS, all of the mice received a single injection of acetaminophen (500 mg/kg). Blood samples were collected 0, 6 and at 24 h, and the mice were sacrificed under ether anesthesia 24 h after acetaminophen injection (n = 10). Hepatic dysfunction was confirmed based on the AST (B) and ALT levels (C) and H&E staining (A). The values shown indicate the means ± S.E.M. *P<0.05 vs. APAP, **P<0.01 vs. APAP, ***P<0.001 vs. APAP.

The mice were injected with a high dose of acetaminophen (500 mg/kg) after PLA2 pre-treatment. Blood samples were collected at 0, 6, and 24 h to measure the AST and ALT levels, and the liver tissues were separated 24 h after the acetaminophen injection for H&E staining. The hepatic cell death in the periportal area was protected by PLA2 injection. In addition, AST and ALT were significantly increased upon acetaminophen administration, and the PLA2 injection significantly suppressed the observed increases in AST and ALT (Fig. 2).

### Hepatoprotective effects of PLA2 in Treg-depleted mice

**Figure 3:**
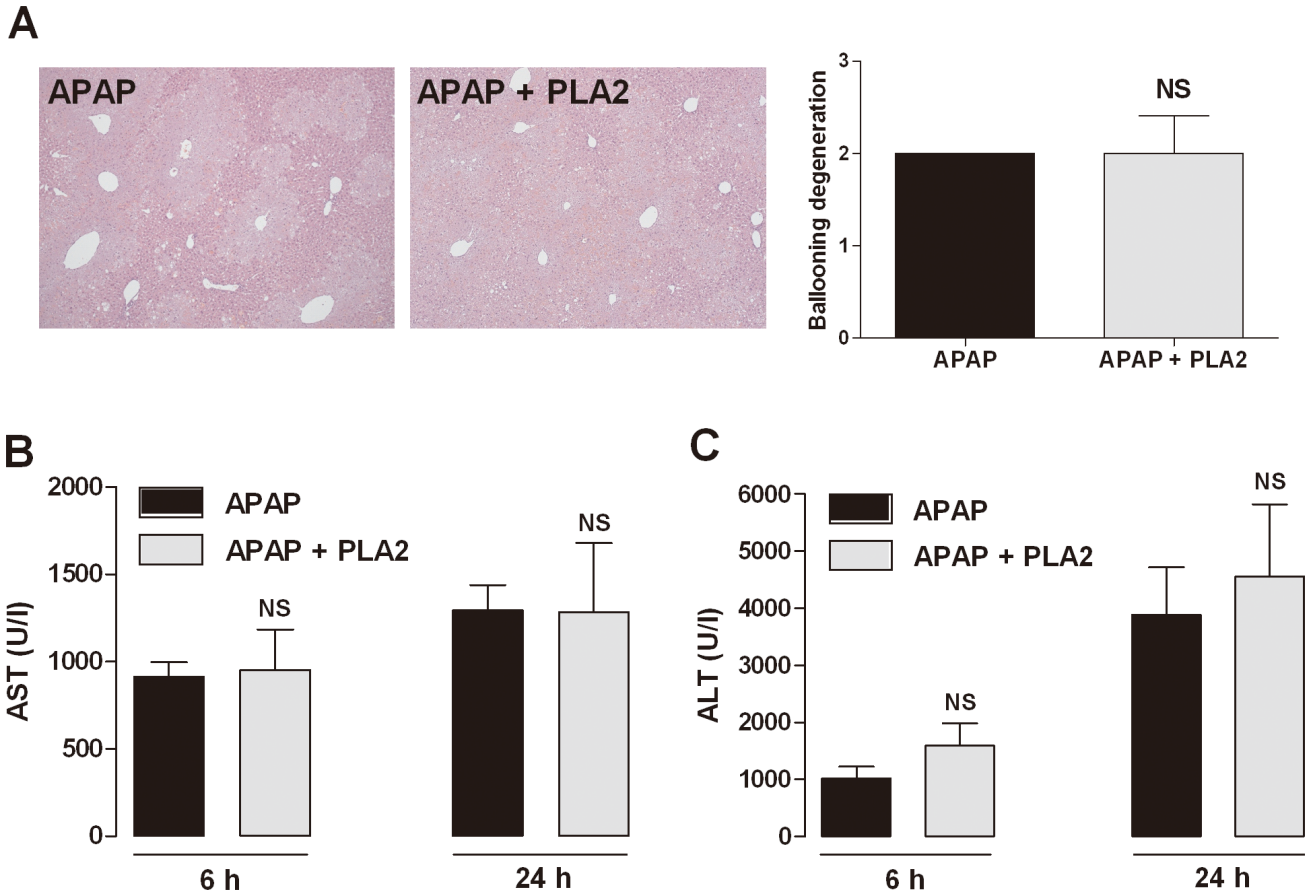
Effect of PLA2 in CD25-depleted mice. To deplete Tregs, an anti-CD25 antibody (0.1 mg/mouse) was injected i.p. each day before the PLA2 and acetaminophen injections. Blood was collected 6 and 24 h after the acetaminophen injection for the measurement of the AST (A) and ALT levels (B) (n = 6). There was no difference between the PBS-treated (APAP) and PLA2-treated (APAP + PLA2) CD25-depleted mice.

To verify whether the effect of PLA2 on acetaminophen-induced hepatotoxicity is mediated by Tregs, we tested the PLA2 effects in a CD4^+^CD25^+^ T cell depletion model by administering mice with an anti-CD25 antibody (0.1 mg/mouse, i.p.). We measured the levels of AST and ALT in the serum. PLA2 treatment had no effect on liver histopathology, AST and ALT level in Treg-depleted mice. The hepatoprotective effects of PLA2 were diminished in Treg-depleted mice. These results demonstrated that Treg depletion eliminated the hepatotoxic protective effects of PLA2 and strongly suggest that the hepatoprotective effects of PLA2 were Treg-dependent (Fig. 3).

### Proinflammatory cytokines in the liver

**Figure 4:**
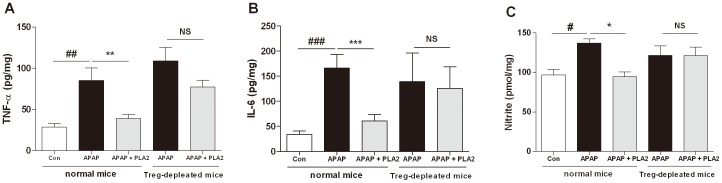
Proinflammatory cytokines in the liver. The proinflammatory cytokines and NO in the liver tissue from normal and CD25-depleted mice were measured by ELISA and the Griess method, respectively. The hepatic TNF (A), IL-6 (B) and NO levels (C) were decreased in the PLA2-treated group (APAP + PLA2) compared with the PBS-treated group (APAP). However, there was no difference between the PBS-treated and PLA2-treated mice in the anti-CD25 model. The values shown indicate the means ± S.E.M. *P<0.05 vs. APAP, **P<0.01 vs. APAP, ***P<0.001 vs. APAP, ^#^P<0.05 vs. Con, ^##^P<0.01 vs. Con, ^###^P<0.001 vs. Con, NS; P>0.05 vs. APAP in anti-CD25 mice (n = 6–8).

To evaluate the antiinflammatory responses of PLA2 treatment in acetaminophen-induced hepatic injury, the levels of TNF, IL-6 and NO in the liver tissue were measured 24 h after acetaminophen injection. Acetaminophen-treated mice exhibited increased levels of TNF, IL-6 and NO. However, the PLA2-treated mice showed significantly lower levels of these inflammatory responses than the control mice, and Treg depletion incapacitated the protective effects of PLA2 (Fig. 4).

### Hepatoprotective effects of PLA2 were mediated by IL-10 production

**Figure 5:**
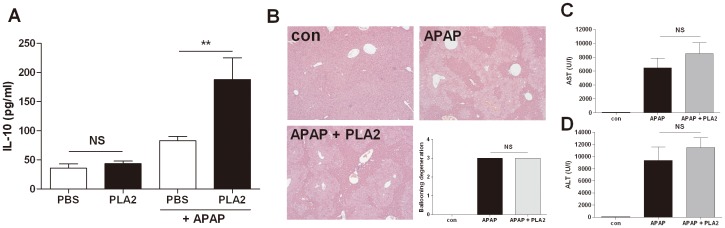
Hepatoprotective effects of PLA2 were mediated by IL-10 production. Mice were injected with acetaminophen (500 mg/kg, i.p.) after PBS or PLA2 injection. Blood samples were collected 6 h after acetaminophen injection (n = 10). IL-10 production in the serum was measured by ELISA (A). IL-10-deficient mice were injected with acetaminophen (500 mg/kg, i.p.) after PBS or PLA2 injection. Blood samples were collected 24 h after acetaminophen injection (B∼D, n = 4). Hepatic dysfunction was reflected by the levels of AST (C) and ALT (D). There was no difference between the PBS-treated (APAP) and PLA2-treated (APAP + PLA2) IL-10^−/−^ mice (B∼D).

It has known that IL-10 protects against acetaminophen-induced liver injury and lethality [21]. PLA2 treatment significantly increased IL-10 production in acetaminophen-treated mice (Fig. 5A). To examine whether the hepatoprotective effect of PLA2 is dependent on IL-10, we used IL-10-deficient mice. We measured the levels of AST and ALT in the serum 24 h after acetaminophen injection. The results showed that the PLA2 effects on acetaminophen-induced hepatotoxicity were abolished in IL-10-deficient mice, suggesting that IL-10 is essential in the PLA2-medicated protective effects in hepatotoxicity (Fig. 5).

## Discussion

The liver is the main organ in the detoxification of drugs and toxins [22]. There are various mechanisms [23], [24] through which drugs may damage the liver [15], [25], [26]. Above all, an overdose of the analgesic drug acetaminophen often causes severe acute hepatotoxicity in experimental animals and humans [27], [28], [29].

In a previous study, Tregs attenuated inflammation in liver injury [30], [31]. The adoptive transfer of Tregs into mice successfully inhibits acute liver injury, whereas the depletion of Tregs aggravated the hepatic toxicity [14]. In our research, we demonstrated that bee venom attenuates inflammatory immune diseases through Treg regulation [32], [33], [34]. In particular, PLA2 from bee venom distinctly increased the Treg population in splenocytes. Based on these results, we hypothesized that PLA2 could have protective effects on acetaminophen-induced hepatotoxicity through Treg modulation.

Fifteen distinct groups of PLA2 have been discovered, and these are categorized into four groups: secreted sPLA2s, cytosolic cPLA2s, calcium-independent iPLA2s, and platelet-activating factor acetyl hydrolase/oxidized lipid lipoprotein-associated PLA2s [35]. Bee venom PLA2 (group III), Indian cobra (group IA) PLA2 and the new world rattlesnake PLA2 (group II) belong to secreted sPLA2. PLA2 is the second most abundant component of bee venom after melittin, and there are many therapeutic effects of bee venom called apitherapy. Although there is a previous report on the hepatoprotective effect of bee venom [36], it did not show which component was responsible for this effect. Other researchers have previously demonstrated the ability of bee venom PLA2 to activate T helper Type 2 cells and the importance of enzymatic activity to this effect [37], [38]. Palm et al., showed that bee venom PLA2 induces the Th2 response through the cleavage of membrane phospholipids and production of lysophospholipids, such as lysophosphatidylcholine [38]. It should be elucidated whether the enzymatic activity of PLA2 is critical for Treg differentiation as a further study.

AST and ALT are secreted into the blood stream in acute liver injury and are important indicators of hepatotoxicity [39], [40], [41]. PLA2-injected mice showed lower levels of AST and ALT in acetaminophen-induced hepatotoxicity compared with PBS-injected mice (Fig. 2). An increased ALT level is associated with hepatic expression of inducible NO synthase (iNOS). The high levels of NO within the liver by iNOS may also promote damage via interference with mitochondrial respiration [42]. NO is an important mediator of acetaminophen-induced hepatotoxicity [43], [44], [45]. PLA2-injected mice exhibited a lower level of NO compared with PBS- treated mice (Fig. 3).

To analyze whether the effect of PLA2 is dependent on Tregs, an anti-CD25 antibody was used to deplete Tregs, and the hepatoprotective effect of PLA2 disappeared as a result. This finding indicates that the hepatoprotective effect of PLA2 is mediated through Tregs. Tregs are associated with the secretion of IL-10 in inflammatory responses [46], [47] and inhibit the secretion of proinflammatory cytokines, such as TNF and IL-6, in liver injury [21], [48]. It has been reported that IL-10 is crucial for tolerance induction in hepatitis and is mainly expressed by Tregs and Kupffer cells. Treg adoptive transfer prevented liver injury, and the depletion of Tregs resulted in reduced plasma IL-10 levels. These findings suggested that Tregs are crucial for primary IL-10 production and augmentation in tolerized mice [14]. It has also been reported that IL-10 protects against acetaminophen-induced liver injury and lethality [21]. Moreover, Louis et al. demonstrated that the administration of recombinant IL-10 protects against hepatotoxicity in a galactosamine and lipopolysaccharide mouse model [49].

Thus, we used IL-10-deficient mice to confirm whether the hepatoprotective effect of PLA2 is dependent on IL-10. The results show that the protective effect of PLA2 was abolished in IL-10-deficient mice (Fig. 5). These results suggest that the protective effect of PLA2 is mediated by IL-10 secretion via Tregs.

The hepatotoxicity of APAP has been attributed to the formation of a highly reactive metabolite N-acetyl p-benzoquinonimine (NAPQI) by the hepatic cytochrome P-450. Substantial amounts of NAPQI are secreted by conjugation with glutathione (GSH). However, in the case of over-dose of APAP, the sulfonation reaction becomes saturated and the over-production of NAPQI depletes GSH in the liver, causing further accumulation of NAPQI. Unconjugated NAPQI binds to proteins and induces cell death that can lead to liver injury [50]. N-acetyl-cysteine (NAC) which is known to stimulate the production of GSH is used as an antidote for overdose of APAP. PLA2 might not have any effects on the APAP metabolism unlike NAC, because protective effects of PLA2 was disappeared in IL-10^−/−^ (Fig 5B∼D) or Treg depleted (Fig 3 and 4) APAP mice suggesting that the hepatoprotective effects of PLA2 were not directly associated with APAP metabolites. It is proposed that PLA2 could be used as an alternative drug for NAC or simultaneously treated with NAC to reduce liver injury by acetaminophen.

In conclusion, PLA2 induces the secretion of IL-10 through Treg modulation and inhibits acute injury in the liver. We suggest that PLA2 has hepatoprotective effects in acetaminophen-induced acute toxicity through modulation of Tregs and IL-10 in mice.

## References

[1] Lee WM (2008) Acetaminophen-related acute liver failure in the United States. Hepatol Res 38 Suppl 1S3–8. 19125949 10.1111/j.1872-034X.2008.00419.x

[2] Liu J, Liu Y, Madhu C, Klaassen CD (1993) Protective effects of oleanolic acid on acetaminophen-induced hepatotoxicity in mice. J Pharmacol Exp Ther 266:1607–1613. 8371159

[3] Patterson AD, Shah YM, Matsubara T, Krausz KW, Gonzalez FJ (2012) Peroxisome proliferator-activated receptor alpha induction of uncoupling protein 2 protects against acetaminophen-induced liver toxicity. Hepatology 56:281–290. 22318764 10.1002/hep.25645 PMC3378765

[4] Wang AY, Lian LH, Jiang YZ, Wu YL, Nan JX (2010) Gentiana manshurica Kitagawa prevents acetaminophen-induced acute hepatic injury in mice via inhibiting JNK/ERK MAPK pathway. World J Gastroenterol 16:384–391. 20082487 10.3748/wjg.v16.i3.384 PMC2807962

[5] Yin H, Cheng L, Holt M, Hail N Jr, Maclaren R, et al (2010) Lactoferrin protects against acetaminophen-induced liver injury in mice. Hepatology 51:1007–1016. 20099297 10.1002/hep.23476 PMC2908515

[6] Zhao X, Cong X, Zheng L, Xu L, Yin L, et al (2012) Dioscin, a natural steroid saponin, shows remarkable protective effect against acetaminophen-induced liver damage in vitro and in vivo. Toxicol Lett 214:69–80. 22939915 10.1016/j.toxlet.2012.08.005

[7] Sakaguchi S, Yamaguchi T, Nomura T, Ono M (2008) Regulatory T cells and immune tolerance. Cell 133:775–787. 18510923 10.1016/j.cell.2008.05.009

[8] Sakaguchi S (2005) Naturally arising Foxp3-expressing CD25+CD4+ regulatory T cells in immunological tolerance to self and non-self. Nat Immunol 6:345–352. 15785760 10.1038/ni1178

[9] Sakaguchi S, Ono M, Setoguchi R, Yagi H, Hori S, et al (2006) Foxp3+ CD25+ CD4+ natural regulatory T cells in dominant self-tolerance and autoimmune disease. Immunol Rev 212:8–27. 16903903 10.1111/j.0105-2896.2006.00427.x

[10] Vignali DA, Collison LW, Workman CJ (2008) How regulatory T cells work. Nat Rev Immunol 8:523–532. 18566595 10.1038/nri2343 PMC2665249

[11] Kobayashi N, Hiraoka N, Yamagami W, Ojima H, Kanai Y, et al (2007) FOXP3+ regulatory T cells affect the development and progression of hepatocarcinogenesis. Clin Cancer Res 13:902–911. 17289884 10.1158/1078-0432.CCR-06-2363

[12] Stross L, Gunther J, Gasteiger G, Asen T, Graf S, et al (2012) Foxp3+ regulatory T cells protect the liver from immune damage and compromise virus control during acute experimental hepatitis B virus infection in mice. Hepatology 56:873–883. 22487943 10.1002/hep.25765

[13] Wang WH, Jiang CL, Yan W, Zhang YH, Yang JT, et al (2010) FOXP3 expression and clinical characteristics of hepatocellular carcinoma. World J Gastroenterol 16:5502–5509. 21086571 10.3748/wjg.v16.i43.5502 PMC2988246

[14] Erhardt A, Biburger M, Papadopoulos T, Tiegs G (2007) IL-10, regulatory T cells, and Kupffer cells mediate tolerance in concanavalin A-induced liver injury in mice. Hepatology 45:475–485. 17256743 10.1002/hep.21498

[15] Bradham CA, Plumpe J, Manns MP, Brenner DA, Trautwein C (1998) Mechanisms of hepatic toxicity. I. TNF-induced liver injury. Am J Physiol 275:G387–392. 9724248 10.1152/ajpgi.1998.275.3.G387

[16] Burke JE, Dennis EA (2009) Phospholipase A2 structure/function, mechanism, and signaling. J Lipid Res 50 SupplS237–242. 19011112 10.1194/jlr.R800033-JLR200 PMC2674709

[17] Monti MC, Casapullo A, Santomauro C, D'Auria MV, Riccio R, et al (2006) The molecular mechanism of bee venom phospholipase A2 inactivation by bolinaquinone. Chembiochem 7:971–980. 16671124 10.1002/cbic.200500454

[18] Zhao H, Kinnunen PK (2003) Modulation of the activity of secretory phospholipase A2 by antimicrobial peptides. Antimicrob Agents Chemother 47:965–971. 12604528 10.1128/AAC.47.3.965-971.2003 PMC149322

[19] Jeong JK, Moon MH, Bae BC, Lee YJ, Seol JW, et al (2011) Bee venom phospholipase A2 prevents prion peptide induced-cell death in neuronal cells. Int J Mol Med 28:867–873. 21701769 10.3892/ijmm.2011.730

[20] Lopez-Vales R, Ghasemlou N, Redensek A, Kerr BJ, Barbayianni E, et al (2011) Phospholipase A2 superfamily members play divergent roles after spinal cord injury. FASEB J 25:4240–4252. 21868473 10.1096/fj.11-183186 PMC3236632

[21] Bourdi M, Masubuchi Y, Reilly TP, Amouzadeh HR, Martin JL, et al (2002) Protection against acetaminophen-induced liver injury and lethality by interleukin 10: role of inducible nitric oxide synthase. Hepatology 35:289–298. 11826401 10.1053/jhep.2002.30956

[22] Bleibel W, Kim S, D'Silva K, Lemmer ER (2007) Drug-induced liver injury: review article. Dig Dis Sci 52:2463–2471. 17805971 10.1007/s10620-006-9472-y

[23] Malhi H, Gores GJ (2008) Cellular and molecular mechanisms of liver injury. Gastroenterology 134:1641–1654. 18471544 10.1053/j.gastro.2008.03.002 PMC2553363

[24] Park BK, Kitteringham NR, Maggs JL, Pirmohamed M, Williams DP (2005) The role of metabolic activation in drug-induced hepatotoxicity. Annual review of pharmacology and toxicology 45:177–202. 10.1146/annurev.pharmtox.45.120403.100058 15822174

[25] Clemens MG (1999) Nitric oxide in liver injury. Hepatology 30:1–5. 10385631 10.1002/hep.510300148

[26] Dey A, Cederbaum AI (2006) Alcohol and oxidative liver injury. Hepatology 43:S63–74. 16447273 10.1002/hep.20957

[27] Olaleye MT, Rocha BT (2008) Acetaminophen-induced liver damage in mice: effects of some medicinal plants on the oxidative defense system. Experimental and toxicologic pathology: official journal of the Gesellschaft fur Toxikologische Pathologie 59:319–327. 18054472 10.1016/j.etp.2007.10.003

[28] Masubuchi Y, Suda C, Horie T (2005) Involvement of mitochondrial permeability transition in acetaminophen-induced liver injury in mice. Journal of hepatology 42:110–116. 15629515 10.1016/j.jhep.2004.09.015

[29] Bedda S, Laurent A, Conti F, Chereau C, Tran A, et al (2003) Mangafodipir prevents liver injury induced by acetaminophen in the mouse. Journal of hepatology 39:765–772. 14568259 10.1016/s0168-8278(03)00325-8

[30] Sennello JA, Fayad R, Morris AM, Eckel RH, Asilmaz E, et al (2005) Regulation of T cell-mediated hepatic inflammation by adiponectin and leptin. Endocrinology 146:2157–2164. 15677756 10.1210/en.2004-1572

[31] Speletas M, Argentou N, Germanidis G, Vasiliadis T, Mantzoukis K, et al (2011) Foxp3 expression in liver correlates with the degree but not the cause of inflammation. Mediators Inflamm 2011:827565. 21772667 10.1155/2011/827565 PMC3136102

[32] Choi MS, Park S, Choi T, Lee G, Haam KK, et al (2013) Bee venom ameliorates ovalbumin induced allergic asthma via modulating CD4+CD25+ regulatory T cells in mice. Cytokine 61:256–265. 23121887 10.1016/j.cyto.2012.10.005

[33] Chung ES, Kim H, Lee G, Park S, Bae H (2012) Neuro-protective effects of bee venom by suppression of neuroinflammatory responses in a mouse model of Parkinson's disease: role of regulatory T cells. Brain Behav Immun 26:1322–1330. 22974722 10.1016/j.bbi.2012.08.013

[34] Kim H, Lee G, Park S, Chung HS, Lee H, et al (2013) Bee Venom Mitigates Cisplatin-Induced Nephrotoxicity by Regulating CD4(+)CD25(+)Foxp3(+) Regulatory T Cells in Mice. Evid Based Complement Alternat Med 2013:879845. 23476708 10.1155/2013/879845 PMC3586478

[35] Schaloske RH, Dennis EA (2006) The phospholipase A2 superfamily and its group numbering system. Biochim Biophys Acta 1761:1246–1259. 16973413 10.1016/j.bbalip.2006.07.011

[36] Darwish SF, El-Bakly WM, Arafa HM, El-Demerdash E (2013) Targeting TNF-alpha and NF-kappaB activation by bee venom: role in suppressing adjuvant induced arthritis and methotrexate hepatotoxicity in rats. PLoS One 8:e79284. 24278124 10.1371/journal.pone.0079284 PMC3835890

[37] Dudler T, Machado DC, Kolbe L, Annand RR, Rhodes N, et al (1995) A link between catalytic activity, IgE-independent mast cell activation, and allergenicity of bee venom phospholipase A2. J Immunol 155:2605–2613. 7544378

[38] Palm NW, Rosenstein RK, Yu S, Schenten DD, Florsheim E, et al (2013) Bee venom phospholipase A2 induces a primary type 2 response that is dependent on the receptor ST2 and confers protective immunity. Immunity 39:976–985. 24210353 10.1016/j.immuni.2013.10.006 PMC3852615

[39] Laskin DL, Gardner CR, Price VF, Jollow DJ (1995) Modulation of macrophage functioning abrogates the acute hepatotoxicity of acetaminophen. Hepatology 21:1045–1050. 7705777

[40] Waters E, Wang JH, Redmond HP, Wu QD, Kay E, et al (2001) Role of taurine in preventing acetaminophen-induced hepatic injury in the rat. Am J Physiol Gastrointest Liver Physiol 280:G1274–1279. 11352821 10.1152/ajpgi.2001.280.6.G1274

[41] Whyte IM, Francis B, Dawson AH (2007) Safety and efficacy of intravenous N-acetylcysteine for acetaminophen overdose: analysis of the Hunter Area Toxicology Service (HATS) database. Curr Med Res Opin 23:2359–2368. 17705945 10.1185/030079907X219715

[42] Moncada S, Erusalimsky JD (2002) Does nitric oxide modulate mitochondrial energy generation and apoptosis? Nat Rev Mol Cell Biol 3:214–220. 11994742 10.1038/nrm762

[43] Gardner CR, Heck DE, Yang CS, Thomas PE, Zhang XJ, et al (1998) Role of nitric oxide in acetaminophen-induced hepatotoxicity in the rat. Hepatology 27:748–754. 9500703 10.1002/hep.510270316

[44] Ito Y, Abril ER, Bethea NW, McCuskey RS (2004) Role of nitric oxide in hepatic microvascular injury elicited by acetaminophen in mice. Am J Physiol Gastrointest Liver Physiol 286:G60–67. 12969830 10.1152/ajpgi.00217.2003

[45] Kuo PC, Schroeder RA, Loscalzo J (1997) Nitric oxide and acetaminophen-mediated oxidative injury: modulation of interleukin-1-induced nitric oxide synthesis in cultured rat hepatocytes. J Pharmacol Exp Ther 282:1072–1083. 9262377

[46] Crispe IN (2003) Hepatic T cells and liver tolerance. Nat Rev Immunol 3:51–62. 12511875 10.1038/nri981

[47] Rubtsov YP, Rasmussen JP, Chi EY, Fontenot J, Castelli L, et al (2008) Regulatory T cell-derived interleukin-10 limits inflammation at environmental interfaces. Immunity 28:546–558. 18387831 10.1016/j.immuni.2008.02.017

[48] Nguyen K, D'Mello C, Le T, Urbanski S, Swain MG (2012) Regulatory T cells suppress sickness behaviour development without altering liver injury in cholestatic mice. Journal of hepatology 56:626–631. 22027577 10.1016/j.jhep.2011.09.014

[49] Louis H, Le Moine O, Peny MO, Gulbis B, Nisol F, et al (1997) Hepatoprotective role of interleukin 10 in galactosamine/lipopolysaccharide mouse liver injury. Gastroenterology 112:935–942. 9041256 10.1053/gast.1997.v112.pm9041256

[50] James LP, Mayeux PR, Hinson JA (2003) Acetaminophen-induced hepatotoxicity. Drug Metab Dispos 31:1499–1506. 14625346 10.1124/dmd.31.12.1499

